# Roles of Fibroblast Growth Factors and Their Therapeutic Potential in Treatment of Ischemic Stroke

**DOI:** 10.3389/fphar.2021.671131

**Published:** 2021-04-22

**Authors:** Confidence Dordoe, Keyang Chen, Wenting Huang, Jun Chen, Jian Hu, Xue Wang, Li Lin

**Affiliations:** ^1^ School of Pharmaceutical Sciences, Wenzhou Medical University, Wenzhou, China; ^2^ Department of Neurology, The Second Affiliated Hospital and Yuying Children’ Hospital of Wenzhou Medical University, Wenzhou, China; ^3^ School of the First Clinical Medical Sciences, Wenzhou Medical University, Wenzhou, China; ^4^ Research Units of Clinical Translation of Cell Growth Factors and Diseases Research, Chinese Academy of Medical Science, Beijing, China

**Keywords:** fibroblast growth factors, stroke, therapeutic potential, theoretical mechanism, clinical application

## Abstract

Stroke is the leading cause of death worldwide, and its treatment remains a challenge. Complex pathological processes are involved in stroke, which causes a reduction in the supply of oxygen and energy to the brain that triggers subsequent cascade events, such as oxidative stress, inflammatory responses and apoptosis, resulting in brain injury. Stroke is a devastating disease for which there are few treatments, but physical rehabilitation can help improve stroke recovery. Although there are very few treatments for stroke patients, the discovery of fibroblast growth factors (FGFs) in mammals has led to the finding that FGFs can effectively treat stroke in animal models. As presented in this review, FGFs play essential roles by functioning as homeostatic factors and controlling cells and hormones involved in metabolism. They could be used as effective therapeutic agents for stroke. In this review, we will discuss the pharmacological actions of FGFs on multiple targets, including their ability to directly promote neuron survival, enhance angiogenesis, protect against blood-brain barrier (BBB) disruption, and regulate microglial modulation, in the treatment of ischemic stroke and their theoretical mechanisms and actions, as well as the therapeutic potential and limitations of FGFs for the clinical treatment of stroke.

## Introduction

Stroke is defined as an acute cerebrovascular disease attributed to sudden rupture of blood vessels in the brain or blockage of blood vessels that prevent blood from flowing to the brain (Campbell and Khatri, 2020). The prevalence of stroke is on the rise, and it has been reported to be one of the most common causes of mortality and morbidity worldwide (Causes of Death Collaborators, 2017), and the number of individuals living with its effects are high due to growing and aging of the population (Stinear et al., 2020). For a long time, intravenous recombinant tissue plasminogen activator (rt-PA, alteplase) has been the exclusive therapeutic drug for acute ischemic stroke. Recent advances in mechanical clot retrieval strategies, such as mechanical thrombectomy for the treatment of large artery stroke, allow effective recanalization and have resulted in improvements in patient outcomes (Prabhakaran et al., 2015; Griessenauer et al., 2018). However, a narrow therapeutic window limits the benefits of these strategies. Moreover, the main strategy for secondary stroke prevention is the use of different pharmacological agents, mainly antiplatelets and anticoagulants, but half of patients have an increased risk of recurrent stroke (Arsava et al., 2016). Stroke has been reported to cause inflammation, and activation of the innate immune system is involved in its pathogenesis (Zuo et al., 2020). Neurorestorative progression in stroke is characterized by neurogenesis, angiogenesis, and synaptic plasticity, which are beneficial for functional recovery (Chen et al., 2019). Newly generated blood vessels increase cerebral flow in the ischemic boundary to supply oxygen and nutrients to the ischemic area to improve neurological function (Beck and Plate, 2009). Therefore, the enhancement of angiogenesis may become a promising therapeutic strategy for ischemic stroke treatment.

The blood-brain barrier (BBB) is composed of endothelial cells (ECs), pericytes, astrocyte end-feet, and a basement membrane. Brain ECs that are connected by tight junction proteins and adhesive proteins (e.g., Occludin, ZO-1, and Claudin-5) protect BBB integrity. Following ischemic stroke, the BBB is acutely disrupted, resulting in secondary brain injury due to cerebral edema and the infiltration of peripheral immune cells into the central nervous system (CNS). Accumulating evidence indicates that targeting the BBB may be a promising therapeutic strategy for the treatment of ischemic stroke (Jiang et al., 2018). On the other hand, the production of damage-associated molecular patterns (DAMPs) by neurons and glial cells are dramatically increased after ischemic stroke. Ischemic stroke induces the activation of astrocytes and microglia, the production of proinflammatory cytokines and chemokines and further exacerbation of tissue damage. Therefore, suppression of proinflammatory cytokine expression or promotion of anti-inflammatory cytokine expression by microglial regulation may be another promising treatment for ischemic stroke (Wang et al., 2020).

Stroke is a neurological disease with poor prognosis. The ultimate goal of stroke treatment is to promote neurological function. The subventricular zone (SVZ) and subgranular zone are known to contribute to functional recovery after stroke through the process of neurogenesis (Rahman et al., 2021). Accumulating evidence suggests that promoting neurogenesis in the chronic phase of ischemic stroke retards disease progression and improves neurological dysfunction (Freret et al., 2006; Bonsack et al., 2020). Therefore, enhancing neurogenesis has become an attractive approach promoting recovery of in the chronic phase of stroke.

There are serious barriers to the clinical translation of drug treatments for stroke. More than 1,000 therapeutic drugs that were shown to have preclinical therapeutic potential for the treatment ischemic brain injury failed in clinical trials (Candelario-Jalil and Paul, 2021). There are still no effective therapeutic strategies that have been shown to improve outcomes after stroke. The complexity of stroke and its associated comorbidities may limit the effectiveness of neuroprotective drugs.

Fibroblast growth factors (FGFs) are polypeptide growth factors involved in numerous processes, such as growth, development, neuronal functions, metabolism, proliferation, migration, apoptosis, wound repair, and angiogenesis (Itoh and Ornitz, 2011). In humans, FGFs support blood vessels to help supply of nutrients to the brain and other organs (Matkar et al., 2017). Similarly, their homeostatic functions enable them to repair tissues, accelerate wound healing and control the nervous system (Beenken and Mohammadi, 2009). The angiogenic and neurotrophic characteristics of FGFs suggest that they may be effective therapeutic agents for ischemic stroke treatment. Many studies have shown that some FGFs are associated with stroke. In this review, we summarize the potential roles of FGFs in promoting neural protection, neuroregeneration, vascular protection, angiogenesis, and BBB protection after ischemic stroke and suggest that FGFs may be candidate agents for improving stroke outcome through multiple pathways.

## FGF Family

The FGF family is made up of signaling and nonsignaling proteins that are structurally related and grouped into 6 subfamilies based on their properties and sequences (Itoh and Ornitz, 2011; Ornitz and Itoh, 2015). The FGF1 subfamily includes FGF1 and FGF2; the FGF4 subfamily comprises FGF4, FGF5, and FGF6; the FGF7 subfamily includes FGF3, FGF7, FGF10, and FGF22; the FGF8 subfamily comprises FGF8, FGF17, and FGF18; the FGF9 subfamily includes FGF9, FGF16, and FGF20; the FGF11 homologous subfamily comprises FGF11, FGF12, FGF13, and FGF14; and the FGF15/19 subfamily includes FGF15/19, FGF21, and FGF23 (Krejci et al., 2009). The FGF family exerts survival-promoting and protective effects to promote neural outgrowth and neurogenesis in the brain (Mudo et al., 2009). Signaling FGFs are expressed in nearly all tissues, and they play roles in embryonic development and organogenesis at the onset of development and function as homeostasis factors for repair, regeneration and maintenance in adults (Ornitz and Itoh, 2001). Nonsignaling FGFs are called intracellular FGFs because they serve as cofactors for the regulation of voltage-gated sodium channels and other molecules, making them essential regulators of neuronal and myocardial excitability (Goldfarb, 2005). The various characteristics of FGF family members and their receptors make them attractive targets for drug development (Belov and Mohammadi, 2013). Members of five other subfamilies, i.e., the FGF1, FGF4, FGF7, FGF8, and FGF9 subfamilies, have paracrine functions; however, FGF9, FGF16, and FGF20 have bipartite signal sequences that are not cleaved (Revest et al., 2000). The table below provides a summary of the FGF subfamilies, their expression sites and their functions (Table 1).

**TABLE 1:** FGF subfamily and their function.

FGF subfamily	Expression sites	Function	References
FGF 1 subfamily			
FGF 1	Brain, pituitary, nerve tissue, retina, adrenal gland, heart, and bone	Promotes mitosis, wound healing, angiogenesis, hematopoiesis, tumorigenesis, and neurogenesis	Cheng et al. (2011), Zou et al., (2020)
FGF 2	Various tissues and organs derived from mesoderm and neuroectoderm, and tumor tissues	Promotes mitosis vascular remodeling, bone formation, pulmonary fibrosis, neurodevelopment, and tumor metabolism	Charoenlarp et al. (2017), Koo et al. (2018)
FGF 4 subfamily			
FGF 4	Posterior part of the limb buds	HST-1; is involved in limb development and internal organs development	Robertson et al. (1997), Okunieff et al. (2003)
FGF 5	Brain	Is involved in hair follicle development, regulates neuronal differentiation and survival and regulates GFAP expression	Lindholm et al. (1994), Reuss et al. (2003)
FGF 6	Developing skeletal muscle	HST-2; is involved in myogenesis and muscle regeneration	de Lapeyriere et al. (1990)
FGF 7 subfamily			
FGF 3	Mammary tumors	Controls the inner ear plan	Dickson et al., (1989)
FGF 7	Fetal lung mesenchymal tissue	KGF; prevents lung branch formation, and lung inflammation	Finch and Rubin (2004)
FGF 10	First observed in the limb bud	Knockout mice, show absence of lungs and complete resection of the fore and hind limbs, promotes the proliferation of mammary gland epithelial cells and reduced apoptosis	Cui and Li (2013)
FGF 22	Mammalian brain and skin wounds	Presynaptic molecule; is involved in repair, stimulates the formation of inhibitory presynaptic terminal, alleviates depression, and is involved in vesicle clustering, and skin development	Beyer et al. (2003), Xu et al. (2017)
FGF 8 subfamily			
FGF 8	Progenitor cells in the midbrain and hindbrain	AIGF; sets up and maintains the midbrain border and regulates the growth and differentiation of progenitor cells to generate midbrain and hindbrain structures	Lim et al. (2015)
FGF 17	Cortex	Has similarities with FGF8; acts as an autocrine growth factor in neoplastic prostate epithelial cells and is involved in neocortex development	Polnaszek et al. (2004)
FGF 18	Skin and cortical neurons	Promotes chondrogenesis, cortical neurons, skin repair, and neuroprotection	Ellsworth et al. (2004)
FGF 9 subfamily			
FGF 9	Neurons in the cortex hippocampus, thalamus, cerebellum, spinal cord, epithelium and mesothelium	Stimulates glial cell growth, is involved in fetal lung development, and enhances the survival of acetylcholinesterase (AChE)-positive neurons	Kanda et al. (2000), Ellsworth et al. (2004), Lum et al. (2009)
FGF 16	Embryonic brown adipose tissue, and the inner ear	Is involved in proliferation of embryonic brown adipose tissue and fate determination of otic cells	Konishi et al. (2000)
FGF 20	Brain	Enhances the survival of midbrain dopaminergic neurons and protects against PD.	Boshoff et al. (2018), Xu et al. (2018)
FGF 15/19 subfamily			
FGF 15	Absorptive cells of the mouse ileum	Is involved in feedback inhibition of hepatic bile acid synthesis and regulates glucose and lipid metabolism	Potthoff et al. (2011), Huang et al. (2018)
FGF 19	Absorptive cells of the human ileum; can be found in the brain, skin, retina, gallbladder, small intestine, kidney and umbilical cord	Acts as a hormone to protect against infarction in response to bile acid absorption, regulates glucose and lipid metabolism, and nonmitogenic effects	Nishimura et al. (1999), Potthoff et al. (2011)
FGF 21	Muscle, liver, islet β-cells in the pancreas and thymus adipose tissue	Plays important role in glucose and lipid metabolism and protects the cardiovasculature in the heart	Chen et al. (2018), Wang et al. (2018)
FGF 23	Bone, lung, brain, heart, muscle and spleen	Regulates phosphate concentration in plasma, decreases absorption and increases the excretion of phosphate	Kendrick et al. (2011), Ix et al. (2012), Zheng et al. (2020)
FGF homologous family			
FGF 11	Neuroblastoma, retinoblastoma and brain tumors	Expression is in ECs by HIF-1α; stimulates capillary-like endothelial tube formation, which is associated with angiogenesis	Lee et al. (2017)
FGF 12	Brain, eye, heart and testis	Contributes to skeletal growth and developmental failure in grade II and III kashin-beck disease (KBD).	Zhang et al. (2016)
FGF 13	Brain and heart	Is involved in neural differentiation in xenopus early development and controls. proliferation and differentiation of skeletal muscle	Lu et al. (2015), Yue et al. (2018)
FGF 14	Adult cerebellum	Regulates intrinsic excitability of cerebellum purkinje neurons	Shakkottai et al. (2009)

## Fibroblast Growth Factors in the Treatment of Stroke

### FGF1 in Stroke

**FIGURE 1:**
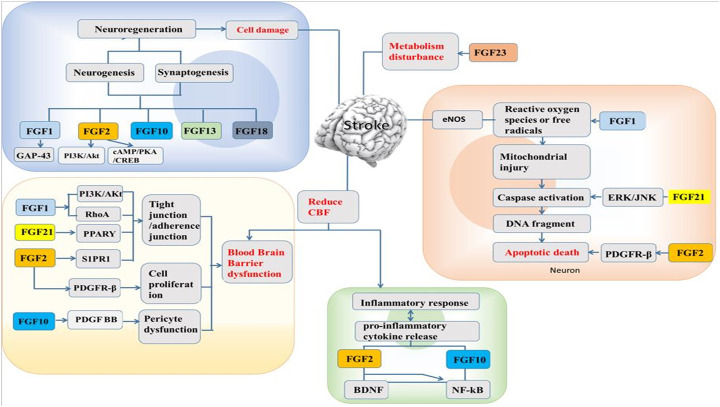
Pharmacological effect of FGFs on stroke and its possible mechanism.

The administration of FGF1 is by a non-invasive method, where it is intranasally delivered into the CNS because it cannot pass through the BBB (Cheng et al., 2011). This intranasal delivery enhanced peripheral nerve regeneration *in vivo* (Jacques et al., 1999) with protective effect against neurofunctional deficit shown to be partially regulated by PI3K/Akt (Wu et al., 2017). Furthermore, FGF1 administered in middle cerebral artery occlusion (MCAO) after 14 days of ischemia insult was seen that, the cerebral infarction area was reduced and the number of blood vessels increased as well (Xu et al., 2009). Intranasal non-mitogenic FGF1 (nm-FGF1) administration for 10 consecutive days enhanced angiogenesis via sphingosine-1-phosphate receptor 1 (S1P1) signaling pathway following stroke (Zou et al., 2020). The mixture of FGF1 and fibrin glue (a slow-release carrier and adhesive agent) in the CNS when applied topically after injury reduced the cerebral infarction, protects cortical cells from loss and reduce microglia penetration thereby promoting a recovery system (Tsai et al., 2015). Treatment with FGF1 preceded in ischemia preserved BBB integrity by regulating tight junctions and adherens junctions’ expressions (Cheng et al., 2011; Chen et al., 2020 **)**. In addition, brain cells with FGF1 exhibited protective effects by upregulating tight junction proteins and inhibiting RhoA through PI3K-Akt-Rac1 pathway regulation to protect the BBB (Wu et al., 2017). From the above, application of FGF1 after stroke create a favorable condition to regulate the injury from neuronal regeneration, neuroprotection, angiogenesis, and BBB protection (Figure 1).

### FGF2 in Stroke

FGF2, also called basic fibroblast growth factor (bFGF), was first purified from the bovine pituitary gland in 1975 (Gospodarowicz, 1975) and is widely expressed in the CNS, especially in the hippocampus (Woodbury and Ikezu, 2014). FGF2 is mainly expressed in neurons and glial cells during puberty and adulthood. It is a polypeptide that has potent trophic and protective effects on the brain and confers neuroprotection after brain injury (Wada et al., 2003). When the expression of FGF2 in the nervous system is high, it shows that FGF2 has neuroprotective effects in animal models of stroke (Kawamata et al., 1997). Reports have proven that FGF2 exerts a neuroprotective effect after stroke (Zhao et al., 2016). FGF2 enhances functional recovery after stroke by promoting progenitor cell proliferation, migration and differentiation in the brain (Bao et al., 2011). Transfection of mesenchymal stromal cells (MSCs) with a herpes simplex virus type 1 (HSV-1) vector expressing FGF2 promotes functional recovery and leads to a reduction in the infarct volume in rats after MCAO (Ikeda et al., 2005). Several other reports have demonstrated a significant reduction in infarct volume in the FGF2-treated group compared to the group that received vehicle treatment only (Ay et al., 1999; Li and Stephenson, 2002). Interestingly, animals given FGF2 show significant improvement, supporting the claim that FGF2 enhances neurogenesis, which contributes to recovery after ischemia (Sun et al., 2009). Platelet-derived growth factor receptor β (PDGFRβ), which is expressed in pericytes and pericyte-derived fibroblast-like cells, plays important roles in the maintenance of the BBB (Hutter-Schmid and Humpel, 2016 **)**. It has been reported that the expression of PDGFRβ is markedly increased in pericytes after ischemic insult (Shibahara et al., 2020). FGF2 increases the number of pericytes to promote pericyte functions via its interaction with PDGF-BB, thus exerting neuroprotective and angiogenic effects (Nakamura et al., 2016). Moreover, injury induces the expression of FGF2 in reactive glial cells to promote synthesis and enhance cell proliferation and neuronal survival; thus, FGF2 has a neuroprotective effect in the brain and maintains the BBB (Wang Z. G. et al., 2016). Sphingosine-1-phosphate (S1P) is a member of sphingolipid family known for regulating cellular processes such as cell growth, angiogenesis and survival (Iwasawa et al., 2018). Following oxygen-glucose deprivation/reperfusion (OGD/R) in human microvascular endothelial cell monolayers, exogenous administration of FGF2 prevents BBB damage by upregulating sphingosine-1-phosphate receptor-1 (S1PR1) protein expression (Lin et al., 2018). Recent studies reported that when the S1P1 is activated, it could ameliorate injuries in MCAO models, and also the S1PR1 modulators involved in S1P1 signaling pathway could restore microvascular circulation to the cerebral ischemia (Li et al., 2019; Zou et al., 2020). In Lin et al. report, they demonstrated the protective ability of recombinant FGF2 on BBB integrity in OGD/R-induced endothelial monolayer permeability, and it was shown that the protective effect of FGF2 was mediated via S1PR1 upregulation (Lin et al., 2018). These results indicate that FGF2 can improve functional recovery and reduce the infarct volume after stroke through various mechanisms. Zhao et al. has demonstrated that FGF2 possess neuroprotective effects through PI3K/Akt activation in a rodent stroke model Zhao et al. (2016). A recent study on the mechanism underlying the effects of cyclic adenosine monophosphate (cAMP)/protein kinase A (PKA) on ischemic injury depends on regulating apoptosis and inflammation, and FGF2-mediated cAMP/PKA/cAMP-response element binding protein (CREB) pathway to promote dendritic and synaptic plasticity (Li et al., 2020). Besides, It has been reported that FGF2 decreased the levels of pro-inflammatory cytokines (IL-6 and TNF-α), through the regulation of the upstream toll-like receptor 4/nuclear factor κB (TLR4/NFκB) signaling pathway (Ye et al., 2015) (Figure 1).

### FGF21 in Stroke

FGF21 is expressed in different tissues and organs, such as the liver, pancreas, skeletal muscle, thyroid and adipose tissue (Nishimura et al., 2000; Watanabe et al., 2020). FGF21 exerts its activity by binding to FGFR and β-klotho (KLB). It has the ability to regulate endocrine processes during glucose and lipid metabolism to aid the production and consumption of energy (Inagaki et al., 2007; Chau et al., 2010). Majority of stroke patients have type 2 diabetes (T2D). Recent studies showed that T2D affects the integrity of BBB and implicates the BBB permeability state after ischemic stroke, but the use of recombinant FGF21 (rFGF21) has been beneficial to the treatment of stroke mice with T2D (Jiang et al., 2020). A report has shown that FGF21 is a suitable mediator for adaptive responses to tissue injury, suggesting it to be a novel therapeutic agent that has a protective ability against stroke stresses in T2D that could improve the neurological outcomes (Jiang et al., 2018). Also, delayed recanalization is another promising alternative for stroke patients who could not meet the window time after stroke injury (Kelly and Holloway, 2018). The delay increases endogenous FGF21 in the penumbra when administered, thereby decreasing the neuronal apoptosis for the enhancement of a better neurological outcome through FGF21/FGFR1/PI3K/caspase-3 signaling pathway (Zheng et al., 2019). Some reports showed that FGF21 plays a significant role in cardiocerebrovascular disease and has the ability to prevent arteriosclerosis by suppressing the hepatic sterol regulatory element (Lin et al., 2015; Wu et al., 2020). A recent report revealed that FGF21 has a low binding affinity for heparan and therefore has a tendency to pass through the BBB (Chen et al., 2018). The peroxisome proliferator-activator receptor gamma (PPARγ) signaling pathway is one of the important downstream pathways of FGF21 and regulates the transcription of genes known to be involved in adipocyte growth and differentiation (Dutchak et al., 2012). Studies have shown that primary neurons expressing FGF21 have a neuroprotective effect against excitotoxicity induced by glutamate (Leng et al., 2015). Lyophilized recombinant human FGF21 (rhFGF21) protects against cerebral ischemia in MCAO rats and neuronal cells by decreasing endoplasmic reticulum (ER) stress (Yang et al., 2018). Ischemia/reperfusion (I/R) was shown to inhibit the activity of endogenous antioxidant enzymes and promote the overproduction of ROS (Lo et al., 2003), ultimately leading to cellular apoptosis (Fleury et al., 2002). Wan et al. found that FGF21 is involved in the signaling pathway responsible for I/R-mediated hippocampal injury (Wan et al., 2019). FGF21 protects against hypoxia stress-induced injury in cerebral microvascular ECs by inducing heat shock protein expression (Wang et al., 2019). Wang et al. found that FGF21 protects against Ang II-induced cerebrovascular aging in ischemia by improving mitochondrial biogenesis and inhibiting p53 activation in an AMPK-dependent manner (Wang X. M. et al., 2016). FGF21 was shown to alleviate MCAO-induced brain injury via activation of PI3K/Akt and inhibition of GSK-3β (Wang et al., 2016). Furthermore, FGF21 decreases the expression of ER stress-related proteins in MCAO rats and PC12 cells (Yang et al., 2018). In addition, in our recent study, we discovered that rhFGF21 treatment alleviates motor nerve dysfunction by modulating microglia/macrophage-mediated neuroinflammation via inhibition of NF-κB signaling pathways (Wang et al., 2020). Overall, FGF21 protects against stroke through actions affecting multiple targets, including promotion of neuronal survival and induction of ER stress and microglia/macrophage-mediated neuroinflammation reductions (Figure 1).

### Other FGFs in Stroke

In addition, other FGFs exhibit potential pharmacological actions against stroke. FGF10 belongs to the FGF7 subfamily and was first cloned in rat embryos (Yamasaki et al., 1996). *In vitro* findings have concluded that exogenous FGF10 is expressed in neurons but not in astrocytes because it is found at levels content in neuronal culture medium and exerts protective effects in neurons deprived of oxygen and glucose (Li et al., 2015). In another study, it was reported that neuron-derived FGF10 can inhibit NF-κB-dependent neuroinflammation and promote neuronal survival by activating the PI3K/Akt signaling pathway in an MCAO mouse model (Li et al., 2016). Comparative genome analysis of stroke-related gene expression profiles has revealed that combination treatments may cause overexpression of the FGF12 gene (Liu et al., 2012). FGF13, which belongs to a homologous FGF subfamily and has a molecular weight of 22 kDa, is distributed widely throughout the developing brain (Lu et al., 2015). Studies have proven that intravenous administration of FGF13 is capable of reducing the infarct volume and brain swelling and alleviating focal cerebral ischemia. The possible mechanism of action of FGF13 may be similar to that of FGF2 (Yao et al., 1999). FGF18 is mainly expressed in the brains of developing embryos (Maruoka et al., 1998). Reports have shown that FGF18 stimulates neurite outgrowth (Ohbayashi et al., 1998) and has mitogenic effects on glial cells and astrocytes (Hoshikawa et al., 2002). Ellsworth and collaborators assessed the neuroprotective effects of FGF18 in MCAO rats and the appropriate treatment time window (Ellsworth et al., 2003; Ellsworth et al., 2004). The results revealed that FGF18 induced a reduction in the infarct volume and improvements in motor ability and exploratory behavior associated with increased cerebral blood flow. FGF18 might be more efficacious than FGF2. FGF18 administration may be effective at both early and later time points. FGF23 is a bone-derived hormone that is expressed in tissues such the lungs, heart, brain, muscles, and spleen and helps ameliorate hyperphosphatemia in patients (Taylor et al., 2011; van Venrooij et al., 2014). A high level of FGF23 in the blood results in a greater risk of cardiovascular disease and stroke (Wright et al., 2014). Previous studies have reported that higher FGF23 expression is associated with a higher risk of stroke (Panwar et al., 2015; Jiang et al., 2016). A health study report revealed that FGF23 is independently associated with a higher risk of stroke (Ix et al., 2012). However, Kendrick’s research and the Northern Manhattan Study (NOMAS) reported opposite findings (Kendrick et al., 2011; Wright et al., 2014). This discrepancy might have resulted from the differences in the associations between FGF23 and stroke subtypes, such as ischemic stroke and hemorrhagic stroke (Yao et al., 2018). Furthermore, higher FGF23 expression exacerbates atherosclerosis (Coban et al., 2018) and toxicity to vessels, leading to the pathophysiology of stroke (Fandler-Höfler et al., 2019). In addition, only one study has focused on FGF7, which exerts a protective effect against ischemic hippocampal neuron damage (Sadohara et al., 2001).

## Progress in FGF Research for Clinical Stroke Treatment

### Therapeutic Potential

Although the therapeutic potential of FGFs in stroke has been indicated by studies in animal models, it has not been reported in the clinic. The main factors that impede the use of FGF in trials is the difficulty in the translation of doses and targeting of the BBB. A phase II/III safety and efficacy trial of FGF2 showed that FGF2 can likely be given safely to stroke patients. The ideal time window for the administration of this agent may exceed 5 h after stroke (Bogousslavsky et al., 2002). However, another study performed in North America was terminated by the sponsor at the advice of an independent data and safety monitoring committee because the incidence of adverse neurological outcomes and mortality was higher in the active treatment groups than the control group (Clark et al., 2000). The most common adverse events associated with FGF2 treatment during and within 2 days after the infusion period were fever, leukocyte activation, vomiting and hypokalemia. Similarly, the intravenous delivery of FGF2 or placebo in the European-Australian phase II/III trial on the 286 acute ischemic stroke patients for more than 24 h showed no significant neuroprotection, but rather caused hypotension and high death rate in treated patients (Bogousslavsky et al., 2002). The basis for the clinical trials of FGF2 were as a result of the improvements shown in the animal models such as the reduction of infarct size, cell proliferation, apoptosis, and improved survival of new mature neurons (Lanfranconi et al., 2011). FGF2 clinical failure raised concerns about the significance of BBB in achieving a remarkable therapeutic level in the brain, and reducing the adverse effects in peripheral tissues. One possible way to consider delivering therapeutic agents to the brain to avoid peripheral side effects is by intranasal administration; thus, a non-invasive method to bypass the BBB into the CNS (Hanson and Frey, 2008). The side effects and mortality rates for the clinical trials of FGF2 were high, and therefore, further experimental investigations are needed to assess the possibility to achieving a pharmacological therapeutic level in the brain, and also focusing on the potential mechanisms of FGF2 delivery outcomes in the pre-clinical trials can be strategy to help researchers determine when to administer treatment to stroke onset. No other studies comparing FGF2 with placebo in patients with acute stroke have been carried out since these studies.

Furthermore, studies were carried out on the association of FGF23 with stroke (Kendrick et al., 2011; Ix et al., 2012), but these studies were limited because they did not examine FGF23 association with other subtypes of ischemic stroke. Kendrick et al. conducted a study and found no significant association of FGF23 with stroke on 43 persons who had advanced chronic kidney disease (CKD) (Kendrick et al., 2011), whereas the study from Heart and Soul reported 36 individuals with higher FGF23 that was associated with higher risk of incident stroke (Parker et al., 2010). The Northern Manhattan Study (NOMAS) conducted on 212 patients also reported no association of FGF23 with ischemic stroke after adjusting the risk of stroke factors (Wright et al., 2014). However, Panwar et al. demonstrated that there was no significant association of FGF23 concentrations with all incident stroke, but in a prespecified analyses, only cardioembolic stroke occurred due to the high level of FGF23 (Panwar et al., 2015), which is consistent with “FGF23 association with ischemic stroke and its subtypes” study by Zheng et al. (Zheng et al., 2020). Nearly two decades passed, and more randomized clinical trials of FGF in patients with acute ischemic stroke are needed (Wahlgren and Ahmed, 2004 **)**. The new goal of treatment may be enhancing functional recovery rather than achieving immediate neuroprotection. Furthermore, artificial FGFR agonists may be useful alternatives to FGF for the treatment of ischemic vascular disease (Ballinger et al., 1999).

### Serum Biomarkers

The use of blood biomarkers for stroke is being increasingly accepted since biomarkers might help neurologists evaluate stroke. Guo et al. demonstrated that the increase in FGF levels is maintained during the first 2 weeks after stroke (Guo et al., 2006). Previous studies have reported that serum FGF levels in patients with acute ischemic stroke are significantly higher than those of patients in the control group (Song et al., 2002; Golab-Janowska et al., 2019). The prognostic value of FGF21 was conducted on patients with acute ischemic stroke. Proteomic analysis of 25 patients revealed that FGF 21 is expressed at lower levels in intracranial blood than in systemic arterial blood (Maglinger et al., 2020). The potential mechanism associated with FGF21 and acute ischemic stroke outcomes has not been fully elucidated due some limitations (Zheng et al., 2021). Firstly, there might be a selection bias and generalizability concern, since other race/ethnics were not part of the study. However, a balance for including and excluding patients based on the baseline characteristics from the China National Stroke Registry (Wang et al., 2011) was of a benefit, because the participants for the study were from China Antihypertensive Trial in Acute Ischemic Stroke (CATIS). Secondly, changes were not detected from the time of hospitalization because the FGF21 levels and blood pressure were taken once at baseline for the participants, resulting in no evidence of data to determine the association of FGF21 levels and acute ischemic stroke (Wang Z. et al., 2011). Lastly, individuals hospitalized for more than 24 h were included in this study which might be a possibility of inaccuracy of FGF21 levels from the onset of stroke, though a report showed that FGFs are still maintained for 3 days after ischemia (Wang et al., 2016).

The prognostic value of the mechanism of higher levels of FGF23 associated with the risk of stroke is still under exploration, but some contributions have been made to help understand its concept from a meta-analysis study. Studies showed that FGF23 has the ability to exert a direct toxic effect on the heart and vessels which could lead to the activation of renin-angiotensin-aldosterone due to the toxicity of FGF23 (Yao et al., 2018), and this could later induce the pathophysiology of stroke and hypertension (Ma et al., 2010). A clinical study revealed that the population with higher FGF23 concentrations have higher occurrence of left ventricle hypertrophy (LVH) (Mirza et al., 2009). Therefore, considering the relation of LVH with elevated arteriosclerosis, it could be partly explained that FGF23 has an effect on stroke risk. Also, the association of FGF23 with ischemic stroke and its subtypes has some limitations, because the methodologies used to measure the FGF23 levels could cause some biasness in the results (Zheng et al., 2020). Bioinformatics analysis of genes revealed that brain FGF9 gene expression levels are increased in stroke patients (Zou et al., 2019). However, a prospective study including 109 stroke patients did not reveal differences between FGF plasma levels at different time points. Therefore, more research on FGF levels in stroke patients and healthy people involving a larger number of samples should be is needed.

## Conclusion and Prospect

As presented in this review, FGFs could be used to treat stroke due to their pharmacological actions on multiple targets, including the ability to directly promote neuronal survival, enhance angiogenesis, protect against BBB disruption, regulate microglia, reduce the infarct size and promote neurological function (Lin et al., 2018; Yang and Torbey, 2020 **)**. Biochemical studies have revealed the mechanisms by which FGFs improve neurological function after ischemic stroke.

The most studied FGFs to date are FGF1 and FGF2. There are some limitations to the application of FGF1, as it cannot cross the BBB to enter the brain and can lead to metastasis and tumorigenesis because of its mitogenic effect. These limitations could be limiting factors to the development of FGF2 as a protective agent affecting multiple targets for the treatment of stroke, and should be carefully considered. Combination therapies involving FGFs have been shown to exert therapeutic effects against stroke via multiple mechanisms in animal experiments but have not yet been applied clinically (Asgharzade et al., 2020).

In this review, we summarized the protective and survival-promoting effects of FGFs in stroke models. Future research on FGFs and the development of FGFs as novel drugs to treat ischemic stroke are needed to improve clinical outcomes and develop a strategy for functional recovery. The safety, efficacy, timing and dose-dependent effects of FGF in animals and patients following stroke need to be determined in the future. Ongoing studies investigating FGF as a new drug target in the ischemic brain will provide novel insights into the role of FGF in the development of stroke pathogenesis and aid in the development of therapies to enhance stroke recovery.
